# Momentum-resolved spin-conserving two-triplon bound state and continuum in a cuprate ladder

**DOI:** 10.1038/s42005-023-01250-9

**Published:** 2023-06-12

**Authors:** Yi Tseng, Eugenio Paris, Kai P. Schmidt, Wenliang Zhang, Teguh Citra Asmara, Rabindranath Bag, Vladimir N. Strocov, Surjeet Singh, Justine Schlappa, Henrik M. Rønnow, Thorsten Schmitt

**Affiliations:** 1 Photon Science Division, Paul Scherrer Institut, Forschungstrasse 111, CH-5232 Villigen PSI, Switzerland; 2 Laboratory for Quantum Magnetism, Institute of Physics, École Polytechnique Fédérale de Lausanne (EPFL), CH-1015 Lausanne, Switzerland; 3 Department of Physics, Friedrich-Alexander-Universität Erlangen-Nürnberg (FAU), Staudtstraße 7, D-91058 Erlangen, Germany; 4 Indian Institute of Science Education and Research, Dr. Homi Bhabha Road, Pune, Maharashtra 411008 India; 5 European X-Ray Free-Electron Laser Facility GmbH, Holzkoppel 4, 22869 Schenefeld, Germany; 6 Present Address: Department of Physics, Massachusetts Institute of Technology, Cambridge, MA 02139 USA; 7 Present Address: Department of Physics, Duke University, Durham, NC 27708 USA

**Keywords:** Magnetic properties and materials, Topological matter

## Abstract

Studying multi-particle elementary excitations has provided unique access to understand collective many-body phenomena in correlated electronic materials, paving the way towards constructing microscopic models. In this work, we perform O *K*-edge resonant inelastic X-ray scattering (RIXS) on the quasi-one-dimensional cuprate ${{{{{{\rm{Sr}}}}}}}_{14}{{{{{{\rm{Cu}}}}}}}_{24}{{{{{{\rm{O}}}}}}}_{41}$ with weakly-doped spin ladders. The RIXS signal is dominated by a dispersing sharp mode ~ 270 meV on top of a damped incoherent component ~ 400-500 meV. Comparing with model calculations using the perturbative continuous unitary transformations method, the two components resemble the spin-conserving *ΔS* = 0 two-triplon bound state and continuum excitations in the spin ladders. Such multi-spin response with long-lived *ΔS* = 0 excitons is central to several exotic magnetic properties featuring Majorana fermions, yet remains unexplored given the generally weak cross-section with other experimental techniques. By investigating a simple spin-ladder model system, our study provides valuable insight into low-dimensional quantum magnetism.

## Abstract (web summary)

Quasi-one-dimensional spin ladders are useful models to study collective many-body phenomena. Here, the authors investigate the excitations of weakly hole-doped cuprate ladders using oxygen K-edge resonant inelastic X-ray scattering revealing fingerprints of spin singlet multi-triplon bound state and continuum modes that are relevant to understanding various collective spin phenomena, such as Majorana fermions.

## Introduction

The quasi-one-dimensional (q-1D) spin ladders are ideal model materials to study collective many-body phenomena and their elementary low-energy excitations with reduced complexity^1,2^. In the even-leg spin ladders, the gapped magnetic excitation spectrum above the rung-singlet ground state comprises dressed spin-one triplet excitations, called triplons^3^, largely resembling the physical properties of quantum *S* = 1 Haldane chains and enabling studies of quantum spin and charge fluctuations in q-1D physics^4,5^. Additionally, serving as the dimensional crossover between the 1D isolated spin-chains and two-dimensional (2D) Heisenberg models, several theories have predicted an exotic collection of ground-state properties in spin ladders, e.g., resonance-valence-bond (RVB) character, modified d-wave superconductivity, spin-charge cooperative stripe order, etc^4–6^. The corresponding experimental observations for these phenomena have been realized in the spin-ladder subsystem of the hybrid chain-ladder materials ${{{{{{\rm{Sr}}}}}}}_{14-{{{{{\rm{x}}}}}}}{{{{{{{\rm{Ca}}}}}}}_{{{{{{\rm{x}}}}}}}{{{{{\rm{Cu}}}}}}}_{24}{{{{{{\rm{O}}}}}}}_{41+{{{{{\rm{\delta }}}}}}}$^7–9^. The crystallographic structure of ${{{{{{\rm{Sr}}}}}}}_{14-{{{{{\rm{x}}}}}}}{{{{{{{\rm{Ca}}}}}}}_{{{{{{\rm{x}}}}}}}{{{{{\rm{Cu}}}}}}}_{24}{{{{{{\rm{O}}}}}}}_{41+{{{{{\rm{\delta }}}}}}}$ consists of alternating layers of edge-sharing CuO_4_ chains, two-leg cuprate ladders, and rock-salt atomic planes from non-magnetic ions (e.g., Sr, Ca, Y, La, etc)^10^. The chain and ladder physics with distinct low-energy properties and temperature scales have been experimentally demonstrated by inelastic neutron scattering (INS)^7,11^.

**Fig. 1:**
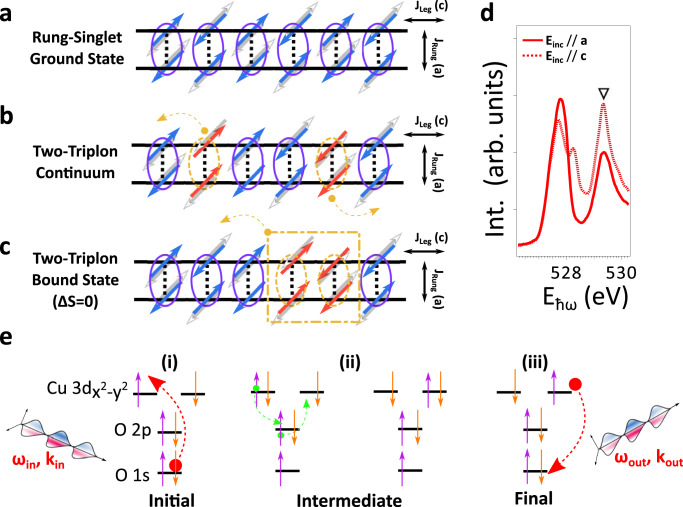
Schematics of two-triplon bound/continuum modes and the spin dynamics generated in O *K*-edge RIXS processes. **a**–**c** Schematics for the spin-singlet ground state, the two-triplon continuum, and the spin-conserving *ΔS* = 0 two-triplon bound state in an undoped two-leg ladder (from top to bottom, respectively). The ground state rung-singlets with fluctuating spin orientations (purple ellipses with blue/gray arrows representing spins pointing in opposite directions) can be excited into the interacting *S* = 1 triplons (orange dashed-line ellipses with spin-triplets, with flipped spins marked as red arrows). This results in either two delocalized triplons moving away from each other that forms the two-triplon continuum or two bound triplons with a total spin *S* = 0, 1, 2. The spin-conserving *ΔS* = 0 two-triplon bound state (orange dashed-dotted line square) is dominating in O *K*-edge resonant inelastic X-ray scattering (RIXS) signal due to the weak spin-orbit interactions of O 1*s* core-levels. Despite these excitations are well studied from the strong (rung-to-leg exchange coupling ratio *r* >> 1) to isotropic (*r* ~1) rung-coupling limit, one should note that the product of rung-singlets is the exact ground state only for the strong rung-coupling cases. **d** O *K*-edge XAS spectra taken at base temperature. The upper Hubbard band resonance (UHB) is marked by the black triangle. **e** Schematics of O *K*-edge UHB RIXS processes. The resultant O 1*s* → Cu ${3d}_{{{{{{{\rm{x}}}}}}}^{2}-{{{{{{\rm{y}}}}}}}^{2}}$ transitions are marked by red dotted-line arrows, which is allowed by the strong hybridization between Cu 3*d* and O 2*p* orbitals that leads to the Cu ${3d}^{10}$ configuration transferring an electron between adjacent sites (green arrows). The unstable local O 1*s* core-hole is subsequently filled by an electron from the Cu 3*d* shell.

A pictorial representation for understanding the interacting *S* = 1 triplons in ladders is shown in Fig. 1, starting from an ensemble of ladder-rung-singlets in the strong rung-coupling ground state (Fig. 1a). With a strong ladder-rung exchange coupling $r={J}_{{{{{{\rm{Rung}}}}}}}/{J}_{{{{{{\rm{Leg}}}}}}}\gg 1$, early mean-field theories demonstrated that the doped ladder excitation spectrum is primarily composed of the singlet-to-triplet gapped triplon^3^, along with minor weight from a holon-spinon coupled mode emerging for light hole-doping^4,5^. When approaching the isotropic limit, where the rung-to-leg exchange coupling ratio *r* ~ 1 as in the ${{{{{{\rm{Sr}}}}}}}_{14-{{{{{\rm{x}}}}}}}{{{{{{{\rm{Ca}}}}}}}_{{{{{{\rm{x}}}}}}}{{{{{\rm{Cu}}}}}}}_{24}{{{{{{\rm{O}}}}}}}_{41+{{{{{\rm{\delta }}}}}}}$ ladder materials, the triplon excitations qualitatively obey the strong rung-coupling limit^1,2^. One should note that the product of rung-singlets is no longer exact despite retaining an approximation for the ground state. Apart from gapped single-triplon excitations, the Heisenberg spin ladder consists of two-triplon composite bound states and delocalized multi-triplon continua that propagate along the ladder-leg direction (Fig. 1b, c).

The understanding of these interacting *S* = 1 ladder triplons has broad significance for low-dimensional or frustrated quantum magnetism. Specifically, the triplon bound states and continua modes bear a similar mathematical formalism with the multi-particle Majorana fermions^12–14^. This was theoretically formulated in the weak rung-coupling regime (*r* << 1), and then recently found to be applicable to the ladder excitation spectra in the vicinity of the isotropic coupling limit (*r* ~ 1)^13,15,16^. Furthermore, such multiple-spin response has been shown to play a significant role in many other quantum material phases, e.g., Higgs modes in 2D Heisenberg models^17^, quadrupolar spin-flip processes in multiferroics, spin-nematic materials^18^, etc. Recent theories further pointed out the possibilities of realizing robust Majorana zero-energy modes in the two-leg Heisenberg *S* = 1/2 spin ladders, providing a rich platform that exhibits great potential for materializing quantum computation^14^. All these signify the importance of understanding the excitations in spin ladders, with their inherent many-body character.

Despite intensive theoretical and experimental studies, the detailed identification of the multi-triplon spectra has been challenging. Recent theories have revealed a series of low-energy excitations based on these ladder triplon modes, and yet their multi-particle nature has posed difficulties in experimental interpretation^15,16,19^. For the real-world synthesized spin-ladder systems or interacting spin-chains, the unbound multi-particle continua extending to high-energy regimes are generally low in scattering cross-section. To simulate such experimental response, it requires accurate theoretical approaches with high-order expansions that consume enormous computation memory^20,21^. Additionally, the total number of bound states varies if magnetic anisotropy or frustrated non-collinear spin exchange are at play as common in most low-dimensional magnetic materials, making it difficult to verify the lowest-lying excitations^20^.

Given these challenges, it is of critical importance to revisit the standard two-leg planar ladders illustrated in Fig. 1. Understanding multi-particle bound states and continua using a non-frustrated two-leg ladder model is advantageous for its simple q-1D geometry. Furthermore, the energy levels for the triplon-triplon scattering have been well treated in theory, and the lowest-lying one- and two-triplon excitations have been shown to dominate the dynamical spectral densities^22^.

The earlier experiments on the two-leg ladder subsystem of ${{{{{{\rm{Sr}}}}}}}_{14-{{{{{\rm{x}}}}}}}{{{{{{{\rm{Ca}}}}}}}_{{{{{{\rm{x}}}}}}}{{{{{\rm{Cu}}}}}}}_{24}{{{{{{\rm{O}}}}}}}_{41+{{{{{\rm{\delta }}}}}}}$ have shown evidence for these triplon excitations. INS measurements are sensitive to the lowest-lying dispersing triplon excitations in the non-spin-conserving *ΔS* = 1 scattering channel^23^. Optical infrared absorption on the hole-depleted cuprate ladders ${{{{{{\rm{Ca}}}}}}}_{14-{{{{{\rm{x}}}}}}}{{{{{{{\rm{La}}}}}}}_{{{{{{\rm{x}}}}}}}{{{{{\rm{Cu}}}}}}}_{24}{{{{{{\rm{O}}}}}}}_{41}$ have revealed the zone-center spin-conserving *ΔS* = 0 two-triplon continuum, and a sharp two-triplon-plus-phonon exciton^24^. The latter was rationalized as the momentum-integrated weight of the *ΔS* = 0 two-triplon bound state that is optically detectable by its coupling to phonons, allowing the required symmetry-breaking for a finite scattering cross-section^25,26^.

In this paper, we study the low-energy excitations in the ladder subsystem of the lightly hole-doped q-1D chain-ladder cuprate ${{{{{{\rm{Sr}}}}}}}_{14}{{{{{{\rm{Cu}}}}}}}_{24}{{{{{{\rm{O}}}}}}}_{41}$ (Sr14) using O *K*-edge resonant inelastic X-ray scattering (RIXS). This two-photon resonant scattering experimental technique is capable of probing various charge-neutral elementary excitations^27^. With the direct access to fluctuating spins at Cu ions, a previous Cu ${L}_{3}$-edge RIXS study has revealed dispersive *ΔS* = 1 two-triplon excitations in Sr14 that were consistent to INS studies^7,28^. Here, we exploit the unique capability of O *K*-edge RIXS at the upper Hubbard band (UHB) resonance for exploring momentum-resolved *ΔS* = 0 magnetic excitations. O *K*-edge RIXS gives access to the *ΔS* = 0 magnetic fluctuations and charge-transfer excitations^29–33^. The potential to detect magnetic excitations beyond two-operator spin-spin correlation functions makes RIXS the ideal probe for investigating the multi-triplon bound and continuum states. This has been shown in recent RIXS reports highlighting contributions from non-locally doubly spin-flipped sites (e.g., higher-order *ΔS* = 0 magnon or spinon continuum; quadrupolar excitations, etc.)^30,33,34^.

We report a sharp dispersing excitation of ~270 meV with comparable energy and momentum dispersion of the collective two-triplon excitations observed by INS and Cu ${L}_{3}$-edge RIXS^23,28^. At higher energy ~400–500 meV, the RIXS signal is dominated by a momentum-independent broad component. Compared with the calculated two-triplon spectral density, the observed excitations ~270 meV and ~400–500 meV are found to resemble the *ΔS* = 0 two-triplon bound state and continuum, respectively^22^. Additionally, we observe a spectral suppression for the dispersive sharp mode ~270 meV upon heating up to ~280 K. The possible scenarios for such spectral melting are briefly discussed.

## Results

### O *K*-edge XAS and UHB RIXS processes

Figure 1d shows the X-ray absorption spectroscopy (XAS) results of Sr14 taken at the O *K*-edge. In this work, we focus on the RIXS spectra probed at the resonance ~529.3 eV, which corresponds to the UHB^35^. The pre-edge double-peak structure of 527.5–528.5 eV gives information on the hole content in the chain and ladder subsystems, respectively^35^. Figure 1e shows the schematics of UHB RIXS processes in cuprates. A photo-excited electron initiated in an O 1*s* → 2*p* transition can hop in between the neighboring Cu ${3d}_{{{{{{{\rm{x}}}}}}}^{2}-{{{{{{\rm{y}}}}}}}^{2}}$ orbitals due to Cu-O hybridization. This can lead to a *ΔS* = 0 final state with two spins at adjacent sites flipped after the de-excitation of the O 2*p* valence electron, which relies on the virtual hopping mediated through Cu-O-Cu superexchange^33^. In step (i), shown in Fig. 1e, an electron is resonantly excited from O 1*s* to O 2*p* states. Then, with the strong hybridization between the O 2*p* and Cu ${3d}_{{{{{{{\rm{x}}}}}}}^{2}-{{{{{{\rm{y}}}}}}}^{2}}$ orbitals, the photo-excited Cu ${3d}^{10}$ electron can effectively hop between adjacent Cu sites with the Cu-O-Cu superexchange shown in step (ii), Fig. 1e. Since the Cu-O hybridization includes the coupling to a short-lived O 1*s* core-hole that is localized, the transferred Cu 3*d* electron through a bridging-oxygen can decay back to fill the unstable core-hole (step (iii) in Fig. 1e). As a result, the UHB RIXS processes give a finite cross-section for the inter-site double spin-flip final state with *ΔS* = 0 (step (iii) in Fig. 1e), as predicted in earlier Cu *K*-edge RIXS studies for low-dimensional cuprates^36,37^. In principle, *K*-edge RIXS involves only weak spin-orbit coupling in the O 1*s* core-levels, and is therefore only sensitive to the *ΔS* = 0 scattering channel^27^. This is in contrast to the *ΔS* = 1 dominant magnetic excitations in cuprates measured by Cu ${L}_{3}$-edge RIXS^27^, which is granted by the strong spin-orbit coupling of Cu 2*p* core-levels, which enable a finite cross-section of the single spin-flip processes in RIXS signal that has been experimentally confirmed in numerous reports^27,38^. Additionally, the detection for high-order multi-triplon continuum modes are granted due to the longer intermediate lifetime scale for O 1*s* core-levels^39^. This gives an enhanced cross-section for the slow double spin-flip excitations, rationalized by the longer intermediate state core-hole lifetime broadening (~150 meV) as compared to the hopping processes at Cu ${L}_{3}$-edge (~300 meV).

### Overview of O *K*-edge RIXS spectra

In this work, the RIXS experimental geometry is fixed to the b-c plane lying in the scattering plane (see Materials and Methods). With this, one can selectively probe the triplon-scattering along ${{{{{\bf{q}}}}}}=({q}_{{{{{{\rm{Leg}}}}}}},{q}_{{{{{{\rm{Rung}}}}}}}=0)$ with predominant low-energy contributions from two-triplon and higher-order modes in even-parity^40^.

**Fig. 2:**
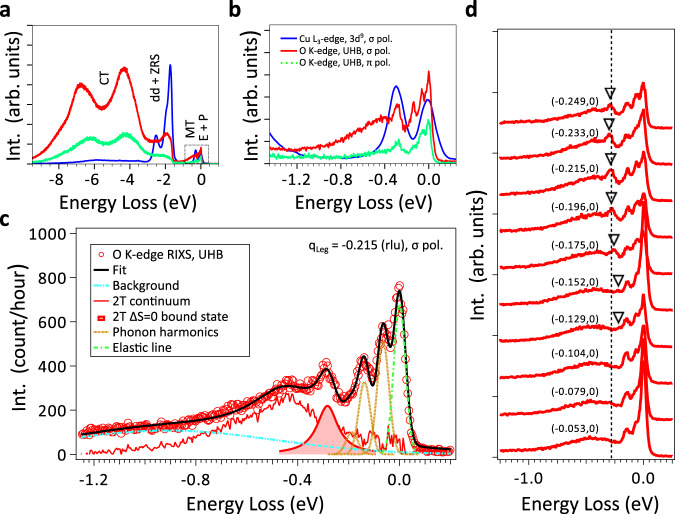
Overview of experimental results. **a** O *K*-edge resonant inelastic X-ray scattering (RIXS) spectra in this work (solid red and dashed green line) compared to the Cu ${L}_{3}$-edge RIXS results provided and re-plotted from a previous study^28^ (solid blue line), with ${{{{{{\bf{q}}}}}}}_{{{{{{\rm{Leg}}}}}}}=-0.215$ (rlu). ${{{{{{\bf{q}}}}}}}_{{{{{{\rm{Leg}}}}}}}$ is the momentum-transfer along the ladder-leg direction expressed in the reduced lattice unit (rlu) of the local Cu-Cu bond distance. The RIXS experiments are performed at base temperature ~20 K. E represents the elastic line that contains a spectral weight of (quasi) elastic scattering and resolution-limited excitations within the instrumental response. P stands for the harmonic satellites of phonon excitations, which we ascribe to be largely contributed by oxygen bond-stretching vibrations. MT denotes the multi-triplon scattering. The inter-orbital transitions of Cu 3*d* shell and the Zhang-Rice singlet exciton are labeled as dd and ZRS, respectively. CT corresponds to the charge-transfer excitations. **b** A zoom to the low-energy region. **c** The spectral assignment with error bars to the fit for the RIXS spectra highlighting the two-triplon (2T) excitations, phonon harmonics and elastic line (see Result subsection “Overview of O K-edge RIXS spectra” and Supplementary Note 1). **d** Momentum-dependent RIXS with ${{{{{\bf{q}}}}}}=({q}_{{{{{{\rm{Leg}}}}}}},{q}_{{{{{{\rm{Rung}}}}}}})$ labeled. Here ${q}_{{{{{{\rm{Leg}}}}}}/{{{{{\rm{Rung}}}}}}}$ is the momentum-transfer along the ladder-leg and rung direction, respectively. Due to the enhanced signal, *σ* polarization is employed for the momentum-dependent RIXS measurements. Black triangles serve as the guide-to-eye for the dispersing sharp peak ~270 meV, while the peak maximum at ${{{{{{\bf{q}}}}}}}_{{{{{{\rm{Leg}}}}}}}=-0.215$ (rlu) is marked by a black dotted line. The “negative” momentum-transfer here indicates that the RIXS spectra are recorded in grazing incidence geometry.

O *K*-edge RIXS spectra of Sr14 are shown in Fig. 2a, b, in comparison with the previous Cu ${L}_{3}$-edge RIXS results^28^. We observe spectral components from charge-transfer (CT) excitations ranging from 3 to 10 eV loss. Around 2 eV loss, the UHB RIXS signal resembles the combined contribution of inter-orbital crystal-field (dd) excitations, and charge-transfer excitons with similar character as Zhang-Rice singlets (ZRS) in 2D cuprates^29,30^. In this work, we focus on the energy regime below 1 eV loss shown in Fig. 2b. The details for spectral fitting shown in Fig. 2c can be found in Supplementary Note 1. Close to the elastic line around 0 eV loss, the weakly-dispersive excitations about ~65 and ~130 meV agree with the two first harmonics of Cu-O bond-stretching phonons in the chains and ladders, as the two subsystems are having similar mode frequencies (see Fig. 2c and Supplementary Note 1)^41,42^. Multi-phonon scattering can be detected as harmonic satellites using RIXS^43^ and a similar spectral response was observed in other q-1D cuprates^44,45^.

Above the phonon modes (extending up to ~150 meV) and below the dd/ZRS excitations (~2 eV), we reveal a sharp peak of ~270 meV on top of a damped spectral component with a maximum of around 400–500 meV. These modes coincide with the energy window of *ΔS* = 0 multi-triplon excitations with exchange coupling ${J}_{{{{{{\rm{Leg}}}}}}}$ ~100–150 meV and electron hopping ${t}_{{{{{{\rm{Leg}}}}}}}$ ~300 meV^15^, consistent with other experimental reports from Sr14^15^. We rule out spectral contributions from the chain subsystem, given their low magnitude of the magnetic exchange coupling ~10 meV^7^ and the localized nature of charge excitations ~1 eV^46,47^. One can see that the broad high-energy component in the O *K*-edge RIXS spectra extends to higher energy loss, compared to the two-triplon profiles in Cu ${L}_{3}$-edge RIXS (Fig. 2b). In Fig. 2d, the momentum-dependent O *K*-edge RIXS measurements further resolve the collective character of these two magnetic excitations. The sharp peak disperses towards lower energy when approaching the zone center, and is then overlapping with the optical phonons for ${{|q}}_{{{{{{\rm{Leg}}}}}}}|$ < 0.13 (rlu). Contrary to this, the energy dispersion for the high-energy damped peak is essentially flat across the momentum-space.

### Experimental observation of *ΔS* = 0 multi-triplon excitations

**Fig. 3:**
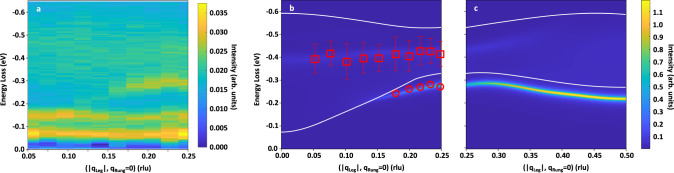
Comparison between experimental spectra and calculated two-triplon spectral densities. **a** Momentum-resolved O *K*-edge resonant inelastic X-ray scattering (RIXS) data taken at base temperature ~20 K. The elastic line is subtracted for clarity. RIXS spectra are normalized to the total integrated intensity from −10 eV loss to 1 eV gain. The normalized RIXS intensity is indicated by the color scale with an upper threshold at 0.035 (arb. units). **b**, **c** Calculated multi-triplon spectral density in the spin-conserving *ΔS* = 0 scattering channel for an undoped ladder using perturbative continuous unitary transformation (pCUT) theory. Subplots are segmented for ${{{{{{\bf{q}}}}}}}_{{{{{{\rm{Leg}}}}}}}$ = [0, 0.25] rlu and [0.25, 0.5] rlu in **b** and **c**, respectively, where **b** covers the experimentally available momentum-space in our O *K*-edge RIXS experiments. ${{{{{{\bf{q}}}}}}}_{{{{{{\rm{Leg}}}}}}}$ is the momentum-transfer along the ladder-leg direction expressed in the reduced lattice unit (rlu) of the local Cu-Cu bond distance. The calculated triplon spectral density is indicated by the color scale with an upper threshold of 1.2 (arb. units). The red circles and squares with error bars are the fitted peak positions for our observed sharp (~270 meV) and broad modes (~400–500 meV) in the RIXS spectra (see respective fits in Supplementary Fig. 1). The white solid line in **b**, **c** curves represents the two-triplon continuum boundary obtained from theoretical calculations. The following parameters are adapted: $r={J}_{{{{{{\rm{Rung}}}}}}}/{J}_{{{{{{\rm{Leg}}}}}}}$ $=0.8$, ${r}_{{{{{{\rm{c}}}}}}}={J}_{{{{{{\rm{Ring}}}}}}}/{J}_{{{{{{\rm{Rung}}}}}}}=0.1$, *J*_Rung_=145 meV. ${J}_{{{{{{\rm{Rung}}}}}}}$, ${J}_{{{{{{\rm{Leg}}}}}}}$, and ${J}_{{{{{{\rm{Ring}}}}}}}$ represent the coupling strength for ladder-rung, ladder-leg, and cyclic ring exchange coupling, respectively. *r* is the ratio between the ladder-rung and the ladder-leg exchange coupling. ${r}_{{{{{{\rm{c}}}}}}}$ is the ratio between the cyclic ring exchange and the ladder-rung coupling. The phonon lines about −65 and −130 meV in **a** are not taken into account in the calculations. In the pCUT theory calculations, the experimental broadening from our RIXS results, e.g., instrumental resolution, spectral overlaps with other excitations, etc, are not implemented.

In the following, we discuss our spectral assignment of the features we observe between 0.2 and 1 eV loss. In Fig. 3, we show the momentum-resolved O *K*-edge RIXS map (Fig. 3a) and simulated *ΔS* = 0 multi-triplon scattering using the perturbative continuous unitary transformation (pCUT) method (Fig. 3b, c). In previous studies, pCUT calculations have successfully captured the collective *ΔS* = 1 triplon excitations of cuprate ladder materials measured by INS, and zone-center *ΔS* = 0 triplon modes observed with optical spectroscopy^23,24,48^. In this study, we calculated the two-quasiparticle processes of an undoped ladder to high order in the *ΔS* = 0 scattering channel, taking rung-triplons as the elementary quasiparticle excitations. The series are obtained in the thermodynamic limit so that no finite-site effects occur and no broadening in the dynamic structure factor is required^22,49,50^. Therefore, theoretical limitations arise from the need of applying extrapolation techniques to the series in order to access the coupling ratio *r* ~1. Regarding the role of doped holes that are absent in our pCUT calculations, we expect potential discrepancies between the calculations and RIXS experiments to be rather small, although two-triplon excitations may mix with the one-triplon scattering upon doping suggested from previous theoretical studies^51^. Nevertheless, the quality of extrapolations has been checked in several works and compared successfully to other numerical approaches^22^ in the parameter regime relevant to Sr14. Indeed, our speculation of predominant *ΔS* = 0 magnetic fluctuations in the 0.2–1 eV RIXS response is supported by the low ladder-hole density of ~6% in Sr14 in line with the XAS results and former experiments^35,52,53^. Specifically, a recent combined experimental RIXS and density matrix renormalization group (DMRG) calculations study also revealed that the calculated spin structure factor for undoped and ~6% hole-doped ladders both resemble well the Sr14 *ΔS* = 1 two-triplon excitations response^54^. Physically, we envision that the average hole distances are sufficiently large at ~6% doping compared to the magnetic correlation length of a few rungs^54^, speaking of a dilute hole distribution such that the triplon mode persists with marginal deviation from the undoped case^55^. We, therefore, expect our RIXS results to mainly correspond to the response of undoped ladders. However, a full treatment of doped Hubbard ladders using numerical approaches such as DMRG is certainly desirable and required to clarify the interplay with the charge carriers and dynamics properly^15,19^. Our assumption is further attested by the reported undoped-like *ΔS* = 1 spin excitation spectra in INS and Cu ${L}_{3}$-edge RIXS^23,28^. Additionally, we adapt an *r* value of 0.8 in our pCUT calculations close to the previously evaluated rung-to-leg ratio of 0.85^15^. More information on the pCUT method can be found in the Materials and Methods.

With the present RIXS experimental geometry at O *K*-edge (Fig. 3a), the momentum-space fraction along ${{{{{{\bf{q}}}}}}}_{{{{{{\rm{Leg}}}}}}}$ can be measured up to $\sim 50 \%$ of the first Brillouin zone (lattice constant ${c}_{{{{{{\rm{Leg}}}}}}} \sim 3.93$ Å)^28^. According to recent theoretical studies, the dispersing sharp mode ~270 meV may have contributions from two types of excitations. One is the *ΔS* = 0 two-triplon bound state and the other one is the holon-spinon mode, both of which can be probed in the spin-conserving *ΔS* = 0 RIXS scattering channel^15^. As for higher spin-transfer modes, the *ΔS* = 1 two-triplon bound state can be resolved in the Cu ${L}_{3}$-edge RIXS cross-section in layered cuprate ladders, while the *ΔS* = 2 two-triplon anti-bound state generally resides in a higher-energy regime and largely overlaps with the two-triplon continuum upper boundary^56^. From strong to isotropic limit, the sizable ladder-rung coupling is expected to grant a spin-singlet two-triplon bound state along ${{{{{\bf{q}}}}}}=({q}_{{{{{{\rm{Leg}}}}}}},{q}_{{{{{{\rm{Rung}}}}}}}=0)$, with energies and momentum dispersion relation close to lowest-lying *ΔS* = 1 two-triplon excitations when *r* ~1^15,16^. This is consistent with our comparison between O K- and Cu ${L}_{3}$-edge RIXS spectra shown in Fig. 2a, b. Near zone-center, the two-triplon bound state mostly overlaps with the two-triplon lower continuum boundary in energies, and is expected to decrease in weight as it could decay into the two-triplon continuum regime (Fig. 3b). Here, the lower boundary of the two-triplon continuum should also exist, and yet the intense bound state dominates in the same momentum and energy regime of our RIXS spectra, while the spectral weight also gets overwhelmed by the broad weight at higher energies ~400–500 meV. For our O *K*-edge RIXS results, the mode energies and momentum dispersions for the dispersing sharp mode ~270 meV are in excellent agreement with the spectral densities of the *ΔS* = 0 two-triplon bound state with ${J}_{{{{{{\rm{Rung}}}}}}} \sim 145$ meV and *r* ~ 0.8, both of which parameters are in line with previous Sr14 studies^15,22^. Our results thus support the prominent contribution of the singlet two-triplon bound state to the ladder *ΔS* = 0 RIXS response, as theoretically predicted^15^.

The other potential contribution for the sharp spectral component ~270 meV is the holon-spinon mode. Optical experiments have shown that Sr14 is lightly hole-doped in the ladder subsystem^57^, where coupled holon-spinons could appear upon doping in Luther-Emery class liquids like even-leg ladders. Recent calculations of the *ΔS* = 0 RIXS response on doped t-J ladders have predicted a dispersing gapless charge mode along ${{{{{\bf{q}}}}}}=({q}_{{{{{{\rm{Leg}}}}}}},{q}_{{{{{{\rm{Rung}}}}}}}=0)$, persisting from the strong to the nearly isotropic regime, that emerges upon hole doping^15^. For Sr14, which is close to the isotropic limit, the predicted energy for this holon-spinon mode would be close to the energy of the dispersing *ΔS* = 0 two-triplon bound state^5,55^. This speaks for a possible contribution from dynamical charge correlations in our O *K*-edge RIXS data^15^. Nevertheless, the holon-spinon weight is found to scale with hole-doping, thus we would expect only a reduced spectral contribution due to the low hole density in the ladder subsystem of Sr14^35,52,53^. Therefore we anticipate that the *ΔS* = 0 RIXS response is dominated here by the spin-conserving magnetic excitations, rather than charge-related fluctuations.

To further elaborate our assessment, we compare our experimental observations to the existing literature reporting signatures of other types of charge excitations in the ladders of the Sr14 compound. We argue that our observed modes are likely not originating from inter-band particle-hole excitations or plasmonic response, both of which were recently revealed by O *K*-edge RIXS in other metal oxides^58,59^. While the optical insulating charge gap ~2 eV is incompatible with the excitation modes of hundreds of meV, our results also did not seem to reproduce any steeply dispersing plasmon despite the experimentally reported plasma edge ~400 meV^57,60^.

For the broad mode ~400–500 meV, we interpret it as the higher-energy two-triplon continuum^15^. The momentum-independent high-energy profiles from RIXS results (Fig. 3a) qualitatively capture the character of the upper boundary of a short-lived two-triplon continuum^15,22^ in pCUT calculations (Fig. 3b). Exact-diagonalization calculations on t-J ladders have predicted appreciable weight for the higher-order continuum states outside the lowest-lying triplon excitations in the *ΔS* = 0 RIXS response^15^. In this energy regime, the spectral contributions from short-lived multi-triplon continua involving more triplons, e.g., the four-triplon continuum contribution^22^, can also be at play. Nevertheless, their spectral weight is expected to be weaker than the two-triplon continuum states. Due to the experimentally damped lineshape, likely due to coupling with higher-order triplon continua or other excitations (e.g., charge dynamics), we compare the spectral centroid of the broad continuum by overlaying it onto the triplon density calculations. Such broad non-dispersive weight extending above 500 meV, similar to the *ΔS* = 0 bimagnon continuum in 2D cuprate reports^29–32^, is absent in the previous Cu ${L}_{3}$-edge RIXS work of Sr14^28^. Similar phenomena were also reported in an O *K*-edge RIXS study on the q-1D spin-chain ${{{{{{\rm{Sr}}}}}}}_{2}{{{{{\rm{Cu}}}}}}{{{{{{\rm{O}}}}}}}_{3}$, where the four-spinon continuum excitations outside the two-spinon continuum boundary were enhanced due to the longer core-hole lifetime for the O 1*s* core-level^33^.

### Temperature-dependent RIXS measurements

Lastly, we extend our analysis of the RIXS observations with temperature-dependent measurements. Finite-temperature effects have been shown crucial for describing the mutual interactions for elementary excitations in q-1D spin systems, as well as their statistical behavior beyond the zero-temperature limit. Upon heating, asymmetric spectral broadening and shifted energy peaks have been inferred in the dynamical structure factor of hard-core bosonic excitations, which turned out to be unexpectedly sensitive to the interplay with available phase space in early theories^61^. Given the high relevance of hard-core bosons in Luther-Emery liquids to the ladder triplons, these studies suggest studying the thermal evolution for understanding the multiple triplon spectra, which has been lacking in current spin-ladder experimental reports.

**Fig. 4:**
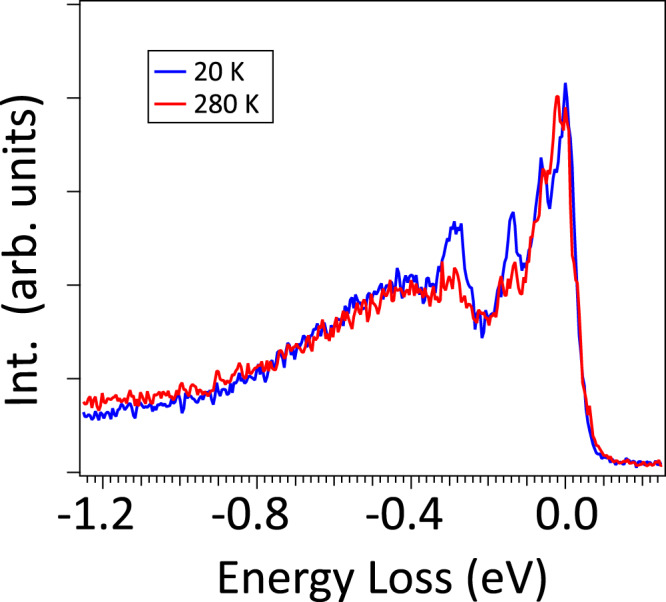
Temperature evolution of the observed spin-singlet dynamics. Temperature-dependent O *K*-edge resonant inelastic X-ray scattering (RIXS) spectra taken with ${{{{{{\bf{q}}}}}}}_{{{{{{\rm{Leg}}}}}}}=-0.215$ (rlu) at 20 K (blue) and 280 K (red). ${{{{{{\bf{q}}}}}}}_{{{{{{\rm{Leg}}}}}}}$ is the momentum-transfer along the ladder-leg direction expressed in the reduced lattice unit (rlu) of the local Cu-Cu bond distance. RIXS spectra are normalized to the total integrated intensity from −10 eV loss to 1 eV gain.

In Fig. 4, the O K-RIXS spectrum at ${{{{{{\bf{q}}}}}}}_{{{{{{\rm{Leg}}}}}}}=-0.215$ (rlu) at ~280 K shows a suppression of the optical phonons and melting of the sharp *ΔS* = 0 two-triplon bound state ~270 meV compared to data taken at base temperature ~20 K. In contrast, the assigned two-triplon continuum at ~400–500 meV shows marginal changes with temperature. We speculate that the observed spectral suppression of the *ΔS* = 0 two-triplon bound state ~270 meV upon heating is connected to the following two mechanisms. Firstly, it has been theoretically shown that the dynamical structure factor in spin ladders exhibits a broadening and suppressed intensity for triplons in the *ΔS* = 1 scattering channel for increased temperatures^61^. This was found to be sensitive to a characteristic temperature scale beyond the triplon gap, similar to our observed temperature evolution of the two-triplon bound state in the *ΔS* = 0 sector. Furthermore, it can be expected that the suppression of the spectral weight is arising from thermal fluctuations that are coupled to triplons. With increasing temperature, the thermally populated triplons were found to transfer their weight from long-lived modes to the rapidly-decaying broad components in the multiple spin-triplet excitations^62^. This would smear out the low-energy triplon density and lead to a spectral broadening beyond the triplon gap energy, which was also observed in INS experiments on Shastry-Sutherland compounds^62^. The other hypothesis is based on former optical studies. Former Raman measurements revealed sharp magneto-optical modes melting across the transition temperature of the charge density wave (CDW) order in isostructural ladders with Sr14^48^. This occurred in an energy window close to our observed *ΔS* = 0 two-triplon bound state. Additionally, recent transport measurements also indicated the emergence of CDW order in the ladders of Sr14 compound melting above ~160 K^63^.

Concerning the spectral melting of the multi-phonon contributions (~65 and ~130 meV) for elevated temperature, we speculate that this originates from a mechanism that suppresses phonon excitations with heating in charge-ordered systems, as recently revealed by RIXS observations of reconstructed momentum- and energy-dependent phonon intensity^64–66^ for different elemental resonances^67^. While former XAS and resonant X-ray scattering studies have revealed a commensurate charge order resonant at the ladder hole contribution^8,35^, the interplay and energy/temperature dependence of the quasi-elastic charge dynamics and phonon excitations by RIXS, as demonstrated in other 2D superconducting cuprates^64–66^, has remained an open issue to our best knowledge. In this regard, we expect our results to motivate further investigations that are required to elucidate the complex electronic degrees of freedom involved in this problem.

## Discussion

We demonstrate the versatile RIXS sensitivity to excitations in different degrees of freedom, and in particular to momentum-resolved *ΔS* = 0 collective spin excitations at the O *K*-edge. In this work, we were taking advantage of the O *K*-edge RIXS processes that were shown to be directly sensitive to not only long-lived *ΔS* = 0 double spin-flip processes effectively probing the two-triplon bound state, but also the rapidly-decaying higher-order *ΔS* = 0 triplon continua in the weakly hole-doped cuprate ladder Sr14. Such spectral capability has been rationalized by the weak spin-orbit coupling and a moderately long core-hole lifetime of O 1*s* core-levels in other q-1D cuprate studies^33,39^.

The experimental observation of *ΔS* = 0 multi-triplon excitations with O *K*-edge RIXS in Sr14 is well described by pCUT calculations for undoped ladders^15,22^. The higher-energy triplon continuum and spin-singlet two-triplon bound state were predicted in the *ΔS* = 0 ladder RIXS signal in recent numerical works based on exact diagonalization^15^. The qualitative agreement between our experimental results and pCUT calculations of the ladder dynamical response^15,22^ facilitates the experimental assignment of these *ΔS* = 0 multi-triplon modes.

Nevertheless, with improved numerical methods for doped Hubbard ladders, such as DMRG including doping explicitly being accessible for large cluster size beyond the exact-diagonalization capability^15^, we believe that future studies utilizing such theoretical framework is of urgent importance to clarify the interplay of charge carrier dynamics with *ΔS* = 0 magnetic multi-triplon response^68^. While the current studies in literature utilizing 2D Hubbard model have tackled the spatial interplay between hole distribution and spin correlations^69,70^, this is still lacking in doped ladders as well as the inclusion of disorder potentials^54^. Furthermore, recent progress in RIXS theories have pointed out the crucial role of including intermediate-state core-hole lifetime effects when calculating the full Kramers-Heisenberg RIXS intensity for narrow resonances like at O 1*s* XAS edge^33^. While this has been demonstrated in doped q-1D cuprate chains using DMRG^71^, it is still lacking for doped ladders. Our work is motivating future theoretical calculations accounting for the exact RIXS cross-section to give deeper insight into the coupled spin and charge dynamics of doped q-1D spin systems.

Additionally, our findings on ladder triplons have implications that are valuable for understanding more complex quantum materials, as well as the potential realization of technical applications. For instance, theories on quantum spin liquid (QSL) systems have suggested that the *ΔS* = 1 and *ΔS* = 0 magnetic excitations are expected to bear fundamentally different responses^72^. While the calculated *ΔS* = 1 sector features incoherent fluxes from fractionalized excitations, the *ΔS* = 0 RIXS signal is dominated by the momentum-dependent Majorana excitations in Honeycomb systems^72^. These Majorana singlet and triplet modes, with distinct effective masses and lifetimes, have been shown to resemble the dispersive triplon bound states and incoherent multi-triplon continua in spin ladders^13,15^. Given the close relation between the ladder triplons and Majorana modes^13^, our results will shed light on the momentum-dependent *ΔS* = 0 spin dynamics that is currently lacking in spin-ladders, and provide an important benchmark for future QSL investigations. On the other hand, RIXS is naturally advantageous for studying micro-mechanical electronics using quantum magnetic materials and their collective spin excitations, which was recently demonstrated in Fe thin films^73^. This is conceptualized as the so-called “magnonics” realm in solid-state material devices, where the transmitted signal is based on charge-free propagation of exchanging spin angular momentum^74,75^. Such conveyed signal reduces heat dissipation and extends its applicability to insulators^74,75^. RIXS capabilities enable the detection of excitation dynamics in various degrees of freedom with various energy-decay channels (e.g., phonons, magnetic or charge continuum modes, etc.), accessible to micron-scale scattering volumes owing to the strong light-matter interactions. Combined with the current state-of-the-art RIXS instruments that can extend to device configurations, our spin-ladder study will leverage the developing magnonics fields with emergent magnetic phenomena and the possibilities of materializing quantum computation with Majorana modes.

## Methods

### Resonant inelastic X-ray scattering (RIXS) experiments

We performed O *K*-edge RIXS and XAS measurements at the ADRESS beamline at the Swiss Light Source, Paul Scherrer Institut^76–78^. The total achieved energy resolution with the RIXS spectrometer operated at the O *K*-edge (~530 eV) was 55 meV. The scattering angle 2θ was fixed at 130°. All measurements were taken at base temperature ~20 K unless specified. Top-post cleavage of the samples was performed in situ at a vacuum pressure of better than $5\times {10}^{-10}$ mbar before all measurements. The RIXS geometry was fixed with the b- and c- axis oriented in the scattering plane as described in the previous Cu ${L}_{3}$-edge RIXS study of ref. ^28^. This will excite the multi-triplon excitations with even-parity that are separated from the odd-parity multi-triplon modes in momentum-space^22,28,40^. By rotating the sample stage with respect to the a-axis (ladder-rungs), we performed momentum-dependent RIXS measurements along the in-plane c-axis (ladder-legs). RIXS measurements were acquired with one hour per spectrum in σ polarization with grazing incidence geometry. XAS spectra were recorded with total fluorescence yield mode. Sr14 single-crystal samples were grown by traveling-solvent floating-zone method with 2–3 mm planar dimension, while the crystallographic orientation was examined by Laue diffraction^79^.

### Two-triplon spectral density calculations: perturbative continuous unitary transformation. (pCUT)

We compare our experimental results to the calculated two-triplon spectral density in the spin-conserving *ΔS* = 0 channel of an undoped two-leg spin ladder using perturbative continuous unitary transformations (pCUT)^22,49,50^. To account for the minimal interactions in an undoped cuprate two-leg spin ladder, we consider the Hamiltonian of a Heisenberg model confined into two-coupled chains, with an additional cyclic four-spin ring exchange as the following^80^:

1 $$H={J}_{{{{{{\rm{Rung}}}}}}}\mathop{\sum }\limits_{{{{{{\rm{i}}}}}}}{{{{{{\boldsymbol{S}}}}}}}_{1,{{{{{\rm{i}}}}}}}{{{{{{\boldsymbol{S}}}}}}}_{2,{{{{{\rm{i}}}}}}}+{J}_{{{{{{\rm{Leg}}}}}}}\mathop{\sum }\limits_{{{{{{\rm{i}}}}}}}{{{{{{\boldsymbol{S}}}}}}}_{{{{{{\rm{\tau }}}}}},{{{{{\rm{i}}}}}}}{{{{{{\boldsymbol{S}}}}}}}_{{{{{{\rm{\tau }}}}}},{{{{{\rm{i}}}}}}+1}+{H}_{{{{{{\rm{cyc}}}}}}}$$

2 $${H}_{{{{{{\rm{cyc}}}}}}}=	 \,{2J}_{{{{{{\rm{Ring}}}}}}}\mathop{\sum }\limits_{{plaquette}}\left[\left({{{{{{\boldsymbol{S}}}}}}}_{1,{{{{{\rm{i}}}}}}}{{{{{{\boldsymbol{S}}}}}}}_{1,{{{{{\rm{i}}}}}}+1}\right)\left({{{{{{\boldsymbol{S}}}}}}}_{2,{{{{{\rm{i}}}}}}}{{{{{{\boldsymbol{S}}}}}}}_{2,{{{{{\rm{i}}}}}}+1}\right)+\left({{{{{{\boldsymbol{S}}}}}}}_{1,{{{{{\rm{i}}}}}}}{{{{{{\boldsymbol{S}}}}}}}_{2,{{{{{\rm{i}}}}}}}\right)\left({{{{{{\boldsymbol{S}}}}}}}_{1,{{{{{\rm{i}}}}}}+1}{{{{{{\boldsymbol{S}}}}}}}_{2,{{{{{\rm{i}}}}}}+1}\right)\right.\\ 	-\left.\left({{{{{{\boldsymbol{S}}}}}}}_{1,{{{{{\rm{i}}}}}}}{{{{{{\boldsymbol{S}}}}}}}_{2,{{{{{\rm{i}}}}}}+1}\right)\left({{{{{{\boldsymbol{S}}}}}}}_{1,{{{{{\rm{i}}}}}}+1}{{{{{{\boldsymbol{S}}}}}}}_{2,{{{{{\rm{i}}}}}}}\right)\right].$$

Here **S** are spin operators with total spin *S* = 1/2. The index i and *τ* runs over rungs and legs (τ = 1, 2) in the two-leg ladder, respectively. J’s represent the antiferromagnetic Heisenberg exchange couplings on rung, legs, and plaquettes of the spin ladder. The spin operations for cyclic ring exchange are summed over every two rungs (a complete square-planar unit), as the two-leg ladders can be seen as a ${{{{{\rm{Cu}}}}}}{{{{{{\rm{O}}}}}}}_{4}$ plaquette-like structure that elongates only along the ladder-leg orientation. Here we utilize the quasiparticle-conserving pCUT method to calculate the one-triplon hopping amplitudes and two-triplon interactions. The continuous unitary transformation is defined as

3 $$\partial {xH}\left(x\right)=[\eta (x),H(x)]$$

mapping the initial Hamiltonian at *x* = 0 to an effective Hamiltonian for x approaching infinity, so that the total number of *S* = 1 triplon quasiparticles is conserved^22,49,50^. η is the infinitesimal anti-Hermitian generator of the transformation defined via the Hamiltonian matrix elements as

4 $${\eta }_{{{{{{\rm{i}}}}}}.{{{{{\rm{j}}}}}}}\left(x\right)={{{{\mathrm{sgn}}}}}\left({q}_{{{{{{\rm{i}}}}}}}-{q}_{{{{{{\rm{j}}}}}}}\right){H}_{{{{{{\rm{i}}}}}}.{{{{{\rm{j}}}}}}}\left(x\right),$$

in the basis of rung eigenfunctions with q being the corresponding eigenvalue^50^.

With this, pCUT generates a series expansion solution of an effective Hamiltonian, which is block-diagonal in the quasiparticle number. To compare with our experimental RIXS response, we calculate the one-triplon hopping amplitudes and two-triplon interactions up to the order of 11 and 10, respectively, by taking the ratio of leg and ring exchange with respect to rung interactions as small perturbations. For the spectral properties of local spin operators, the representative observables O have to be transformed with the same unitary transformation:

5 $$\partial {xO}\left(x\right)=\left[\eta \left(x\right),O\left(x\right)\right].$$

For the *ΔS* = 0 scattering channel, we target the local spin operators that generate a total spin of 0 defined as^81^

6 $$O\left(m,i\right)={{{{{{\boldsymbol{S}}}}}}}_{{{{{{\rm{m}}}}}},{{{{{\rm{i}}}}}}}{{{{{{\boldsymbol{S}}}}}}}_{{{{{{\rm{m}}}}}},{{{{{\rm{i}}}}}}+1},$$

where the index m decides which leg the spin observable operates on.

Before comparing our RIXS results to the calculated triplon density using pCUT, it is necessary to parametrize the magnetic exchange couplings in Sr14. The magnetic excitations of spin ladders are sensitive to the rung-leg ratio r of exchange couplings^13,15^. The weakly hole-doped two-leg ladder system Sr14 was previously identified as an intermediate rung-coupling system with $r={J}_{{{{{{\rm{Rung}}}}}}}/{J}_{{{{{{\rm{Leg}}}}}}} \sim$ 0.85^15^. This is determined by evaluating the local distance of the nearest Cu-O-Cu bonds that accounts for the predominant superexchange coupling^79^. In this work, we adapt an *r*-value of 0.8 for an undoped ladder in the calculations. Additionally, the four-spin cyclic ring exchange interactions ${r}_{{{{{{\rm{c}}}}}}}={J}_{{{{{{\rm{Ring}}}}}}}/{J}_{{{{{{\rm{Rung}}}}}}}$ are included since it is expected to be the dominant corrections of the nearest-neighbor Heisenberg interactions in the square-planar copper-oxide environments. Former studies on cuprate ladders have found that the inclusion of ${J}_{{{{{{\rm{Ring}}}}}}}$ is needed for a quantitative description, in particular, to simultaneously fit the low-energy one-triplon gap and the (higher-energy) dispersion of the *ΔS* = 1 two-triplon bound state^22^. A ring exchange of ~10–20 % of ${J}_{{{{{{\rm{Rung}}}}}}}$ for nearly isotropic ladders was predicted to significantly modify the triplon excitation spectrum, and frustrate the dispersing triplon bound states^22^. For this study, we find that the choice of ${J}_{{{{{{\rm{Ring}}}}}}}$ $=0.1{J}_{{{{{{\rm{Rung}}}}}}}$ is in agreement with the current experimental reports on Sr14 and isostructural compounds^22^, and matches our RIXS results. (see Supplementary Note 2 and Supplementary Fig. 2).

### Supplementary information

Supplementary information

## Data Availability

All the experimental data and numerical results used in the present manuscript and supplementary information can be found at 10.5281/zenodo.7850810.

## Code availability

The code supporting this study will be made available upon request.
