# Supplementary material for: Momentum-resolved spin-conserving two-triplon bound state and continuum in a cuprate ladder
Source: Commun Phys. 2023 Jun 12;6(1):138. doi: 10.1038/s42005-023-01250-9 (PMC11041747; doi:10.1038/s42005-023-01250-9)
Supplement: Supplementary file 1 — Supplementary information [file 42005_2023_1250_MOESM1_ESM.pdf]

Supplementary information for
"Momentum-resolved spin-conserving two-triplon bound state and continuum in a cuprate ladder"

Yi Tseng,[1,2,*,§] Eugenio Paris,[1] Kai P. Schmidt,[3] Wenliang Zhang,[1] Teguh Citra Asmara,[1]
Rabindranath Bag,[4,†] Vladimir N. Strocov,[1] Surjeet Singh,[4] Justine Schlappa,[1,5]
Henrik M. Rønnow,[2] and Thorsten Schmitt[1,*]

[1]*Photon Science Division, Paul Scherrer Institut, Forschungstrasse 111, CH-5232 Villigen PSI, Switzerland.*
[2]*Laboratory for Quantum Magnetism, Institute of Physics, École Polytechnique Fédérale de Lausanne (EPFL), CH-1015 Lausanne, Switzerland.*
[3]*Department of Physics, Friedrich-Alexander Universität Erlangen-Nürnberg (FAU), Staudtstraße 7, D-91058 Erlangen, Germany.*
[4]*Indian Institute of Science Education and Research, Dr. Homi Bhabha Road, Pune, Maharashtra 411008, India.*
[5]*European X-Ray Free-Electron Laser Facility GmbH, Holzkoppel 4, 22869 Schenefeld, Germany.*

*Corresponding authors. Email: tsengy@mit.edu (Y.T.); thorsten.schmitt@psi.ch (T.S.)

Supplementary Note 1: Spectral fitting for O K-edge RIXS

In Supplementary Figure 1, we show the multi-peak fitting for the O K-edge resonant inelastic X-ray scattering (RIXS) spectra measured at the upper Hubbard band (UHB) resonance. The elastic line in the momentum-dependent O K-edge RIXS map (Figure 3 in main text) of the data is subtracted with a Gaussian resolution function of the same spectral intensity as the elastic peak. Due to the changing recognizable number of peaks at different momentum-transfer points, a global fitting across all measured momentum-transfer points is not conducted. We apply the following fitting procedure for $q_{\mathrm{Leg}} = -0.175, -0.196, -0.215, -0.233$ and -0.249 (rlu) for the O K-edge RIXS data, where the sharp peak ~ 270 meV is clearly disentangled from the lower-energy phonons and the higher-energy broad mode. In Supplementary Figure 1, the elastic line and phonon excitations are well fitted by resolution-limited Gaussians. Three equally-spaced satellites of mode energy about 65~70 meV are taken as the initial parameters for fitting the optical phonons in our RIXS spectra. The mode energies and intensities of high-order phonon overtones, up to the third order, are progressively deviating from the ideal harmonic oscillator. This possibly originates from anharmonic interactions in the lattice, or differences in the momentum-dependent electron-phonon coupling for the different phonon modes probed [1]. For $|q_{\mathrm{Leg}}| > 0.13$ rlu, the dispersing sharp peak is fitted with a Lorentzian function. As for the high-energy weakly-dispersive component $\sim 400-500$ meV, we evaluate the peak position by taking the centroid up to 80% of the maximum intensity for the residual weight after subtracting the elastic line, phonons, dispersing sharp peak and a higher-energy background. This was previously shown effective in evaluating the broad charge excitations in doped cuprates using O K-edge RIXS [2]. We choose a Gaussian profile for the background with fixed peak position and width. As the dispersive sharp peak ~ 270 meV cannot be recognized for $|q_{\mathrm{Leg}}| < 0.13$ (rlu), the center of gravity for the high-energy broad mode is evaluated without considering the sharp mode when the approaching zone center.

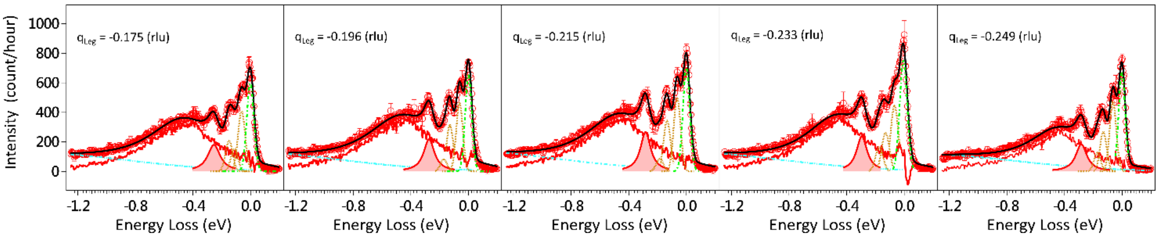

Supplementary Figure 1: Multi-peak fitting for the O K-edge UHB RIXS spectra at selected momentum-transfer points, where the $\Delta S = 0$ two-triplon bound state is clearly separated from the optical phonons and two-triplon continuum.

Supplementary Note 2: Spectral density calculations for $\Delta S = 0$ two-triplon scattering density using perturbative continuous unitary transformation with ring exchange interactions

Here we show the perturbative continuous unitary transformations (pCUTs) calculations with and without the inclusion of four-spin cyclic ring exchange on top of the nearest-neighbor exchange interactions in an undoped ladder Hamiltonian. It has been shown in former studies that a $\sim 10 - 20$ % of ring exchange, scaled by the ladder-rung coupling ($r_c = J_{\mathrm{Ring}}/J_{\mathrm{Rung}}$), better captured the observed triplon excitations in neutron and optical experiments [3]. Generally, the inclusion of cyclic ring exchange leads to a reduced one-triplon gap, and a closer energy in between multi-triplon continuum states (scaled by J_{Rung}) [3].

At the zone center, the two-triplon band minimum at ($q_{\mathrm{Leg}} = 0, q_{\mathrm{Rung}} = 0$) is expected to possess an mode energy of twice the one-triplon gap at ($q_{\mathrm{Leg}} = \pi, q_{\mathrm{Rung}} = \pi$) [4]. This is required given the symmetry operations for triplon spin-spin permutations in an undoped ladder [4]. We thereby take this criteria to quantify our RIXS results from the calculated triplon densities, in combination with the spectral characteristic of triplon bound state dispersions and continuum states that govern the predominant magnetic exchange energies.

In Supplementary Figure 2, we demonstrate the calculated $\Delta S = 0$ triplon densities for the even-number triplon scattering along $\mathbf{q} = (q_{\mathrm{Leg}}, q_{\mathrm{Rung}} = 0)$, with 0 and 10% of ring exchange of ladder-rung coupling, respectively. We adapt the exchange parameters that fit to our RIXS results as well as the former experimental studies. The rung-to-leg ratio r is kept fixed at 0.8 as in the main manuscript. By incorporating the ring exchange, the overall momentum-space spectral profile changes marginally. The calculated two-triplon minimum at $q_{\mathrm{Leg}} = 0$ decreases and evolves towards the speculated value of twice one-triplon gap energy, where the trend is consistent with previous neutron and optical experiments [3].

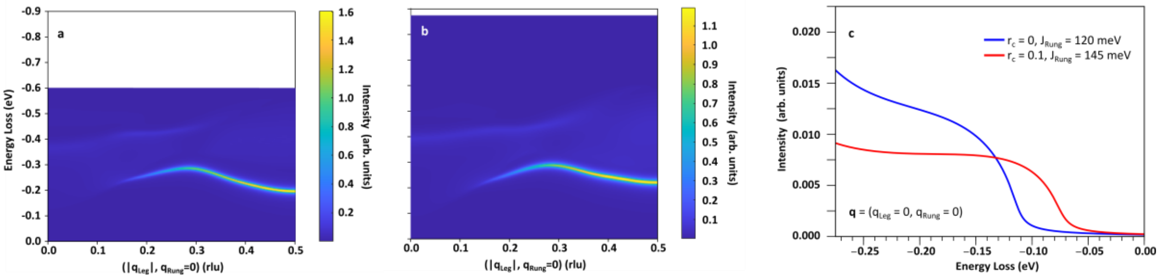

Supplementary Figure 2: Two-triplon spectral density calculations in the $\Delta S = 0$ scattering channel using pCUT with (**a**) $r = 0.8$, $r_c = 0$, $J_{\mathrm{Rung}} = 120$ meV, (**b**) $r = 0.8$, $r_c = 0.1$, $J_{\mathrm{Rung}} = 145$ meV. The spectral intensities are calculated up to 5 and 6 units of J_{Rung} in energy loss for (**a**) and (**b**), respectively. Spectral profiles at ($q_{\mathrm{Leg}} = 0, q_{\mathrm{Rung}} = 0$) for both $r_c = 0$ and $r_c = 0.1$ are shown in (**c**). By monitoring the spectral intensity at zone-center, the lowest-lying downturn with onset around ~ 110 and ~ 70 meV for $r_c = 0$ and $r_c = 0.1$, respectively, where the latter is closer to twice of the one-triplon gap reported in Sr14 (~ 32 meV)[5].

Supplementary References

1. Vale, J. G. *et al.* High-resolution resonant inelastic x-ray scattering study of the electron-phonon coupling in honeycomb α-Li_2IrO_3. *Phys. Rev. B* **100**, 224303 (2019).
2. Ishii, K. *et al.* Observation of momentum-dependent charge excitations in hole-doped cuprates using resonant inelastic x-ray scattering at the oxygen K edge. *Phys. Rev. B* **96**, 115148 (2017).

3. Schmidt, K. P. & Uhrig, G. S. Spectral properties of magnetic excitations in cuprate two-leg ladder systems. *Mod. Phys. Lett. B* **19**, 1179–1205 (2005).
4. Windt, M. *et al.* Observation of two-magnon bound states in the two-leg ladders of (Ca, La)$_{14}$Cu$_{24}$O$_{41}$. *Phys. Rev. Lett.* **87**, 127002 (2001).
5. Eccleston, R. S. *et al.* Spin dynamics of the spin-ladder dimer-chain material Sr$_{14}$Cu$_{24}$O$_{41}$. *Phys. Rev. Lett.* **81**, 1702 (1998).
